# Case Report: Chilblains-like lesions (COVID-19 toes) during the pandemic - is there a diagnostic window?

**DOI:** 10.12688/f1000research.24766.2

**Published:** 2020-08-26

**Authors:** Joanna Ludzik, Alexander Witkowski, Donna E. Hansel, Philipp W. Raess, Kevin White, Sancy Leachman

**Affiliations:** 1 Department of Dermatology, Oregon Health & Science University, Portland, Oregon, 97239, USA; 2 Department of Telemedicine and Bioinformatics, Jagiellonian University, Krakow, 31-008, Poland; 3 Department of Pathology and Laboratory Medicine, Oregon Health & Science University, Portland, Oregon, 97239, USA

**Keywords:** coronavirus, Covid-19, pernio, chilblains, dermoscopy, confocal, diagnostic window

## Abstract

The COVID-19 outbreak caused by the novel coronavirus, SARS-CoV-2, typically presents with symptoms including fever, cough, headache, myalgia, asthenia, anosmia, diarrhea, and sometimes pneumonia, which can be fatal.  Recently, new dermatologic findings have been described in association with the disease that can potentially be a distinguishing feature of infection. One such feature resembles chilblains and this case report represents a presentation of this feature with a 48-year-old female with violaceous lesions with surrounding pink erythema on her toes who tested negative for COVID-19.

## Introduction

The COVID-19 outbreak caused by the novel coronavirus, SARS-CoV-2, typically presents with symptoms including fever, cough, headache, myalgia, asthenia, anosmia, diarrhea, and sometimes pneumonia, which can be fatal. Recently, new dermatologic findings have been described in association with the disease that can potentially be a distinguishing feature of infection. One such feature resembles chilblains ^1^. Chilblains-like lesions, described in multiple case reports from around the globe, have demonstrated either an erythematous-edematous or blistering skin lesion that mostly affects the toes and soles. Over the course of one to two weeks, lesions become more purpuric and flatten, finally resolving spontaneously without any treatment. The majority of patients with chilblains-like lesions are generally in good health, without significant coronavirus symptoms, may have a recent history of mild upper respiratory symptoms, but no prior history of similar cutaneous lesions ^2^. By contrast, non-COVID-19-related chilblains are associated with exposure to low temperatures and may be associated with autoimmune disorders (chilblain lupus), hematologic disorders and rarely viral infections. During recent months, chilblains-like lesions has been reported in association with COVID-19, although the timing of these lesions relative to active infection appears to vary. In a cohort of patients reported from Italy, some patients developed skin findings only during the initial course of disease, while others reported onset at later stages ^3^.

The ill-defined timing of presentation of chilblains-like lesions in confirmed COVID-19 positive patients may be associated with onset, progression or resolution of the disease. Due to potentially unreliable and subjective patient reporting, lack of awareness that these lesions may be a symptom of COVID-19 disease, and limited coronavirus testing availability, the diagnosis of COVID-19 and/or chilblains-like lesions may be missed or unreported. Based on our experience with the COVID-19 population in Oregon, we hypothesize that there may be a limited and possibly non-overlapping diagnostic window for COVID-19 infection and chilblains-like lesions. If true, this could result in classic chilblains-like lesions features in the absence of a positive COVID-19 diagnostic test. Here, we present the case of a 48-year-old healthy woman, who presented with chilblains-like lesions on her toes and tested as negative for COVID-19.

## Case report

**Figure 1.**
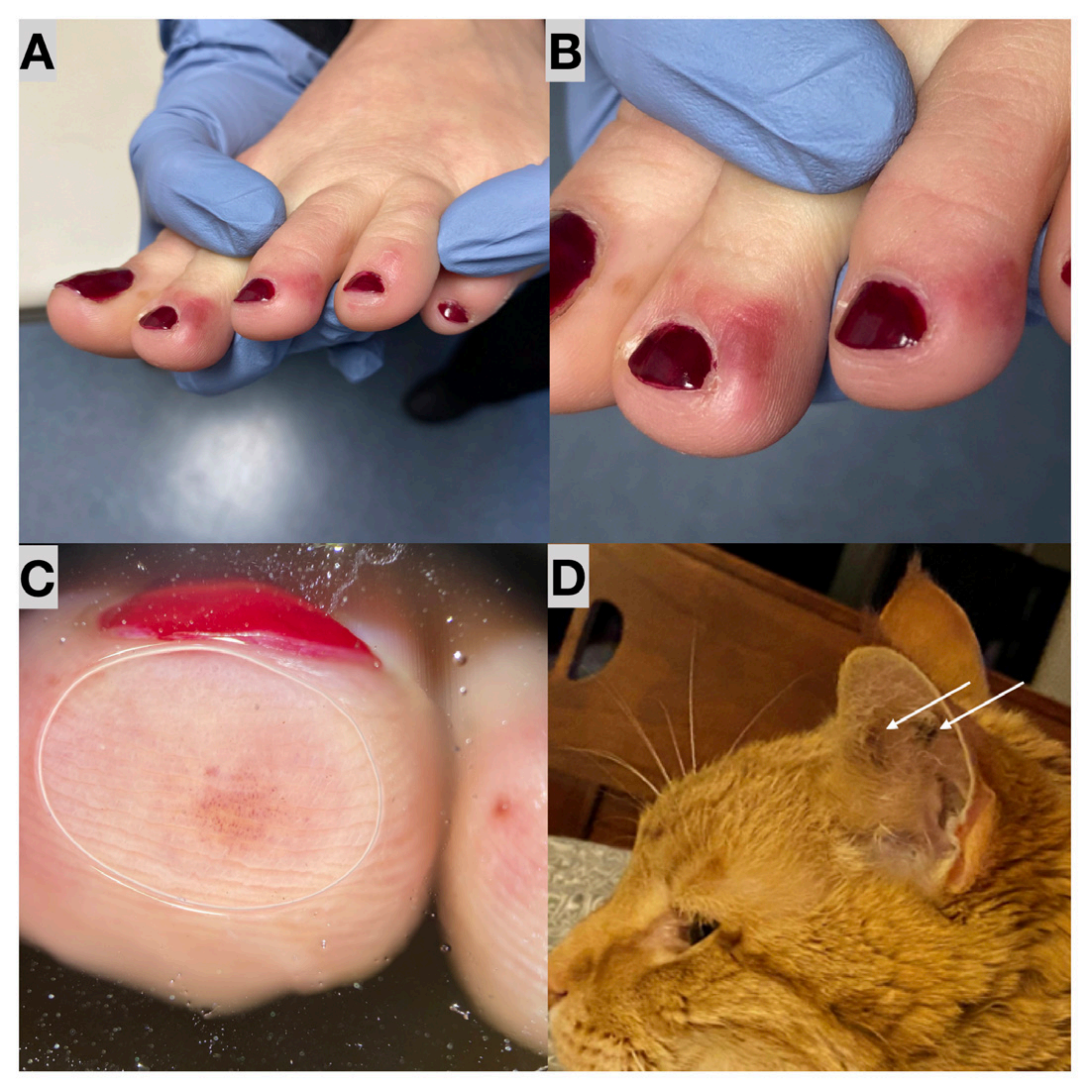
Initial presentation of patient and her pet cat. ( **A**) Clinical images taken of the patient’s left foot, showing multiple well circumscribed violaceous discolorations with surrounding pink erythema on the distal toes. ( **B**) Closeup of the chilblains-like lesions. ( **C**) Dermoscopy image of the fourth toe showing multiple red globules. ( **D**) Patient-submitted clinical photos of her indoor pet cat with multiple red-purple well-circumscribed slightly raised spots bilaterally that also resolved spontaneously. Additionally, ocular discharge was present.

A 48-year-old healthy female patient presented to our hospital with chilblains-like lesions on her toes. She had no other underlying diseases and denied recent exposure to cold temperatures. She is a healthcare provider with potential exposure to SARS-CoV-2. The patient reported upper respiratory symptoms four weeks prior to development of lesions, which included mild sore throat, non-productive cough, and chest pain. A viral test was unavailable at that time. Her highest temperature was 99.4°F. Concurrent with the onset of cutaneous manifestations, she and her husband both experienced intermittent diarrhea over a period of 3 days without any recurrent respiratory symptoms or fever. Two 9-year old children in the home were asymptomatic. The patient began developing small blisters on both her feet, starting with a single, isolated lesion on the bottom of one toe. Additional lesions developed on the lateral aspects and top of 6 toes, primarily at the distal aspect. The cutaneous lesions slowly progressed from pink macules and papules to violaceous lesions with surrounding pink erythema ( Figure 1).

**Figure 2.**
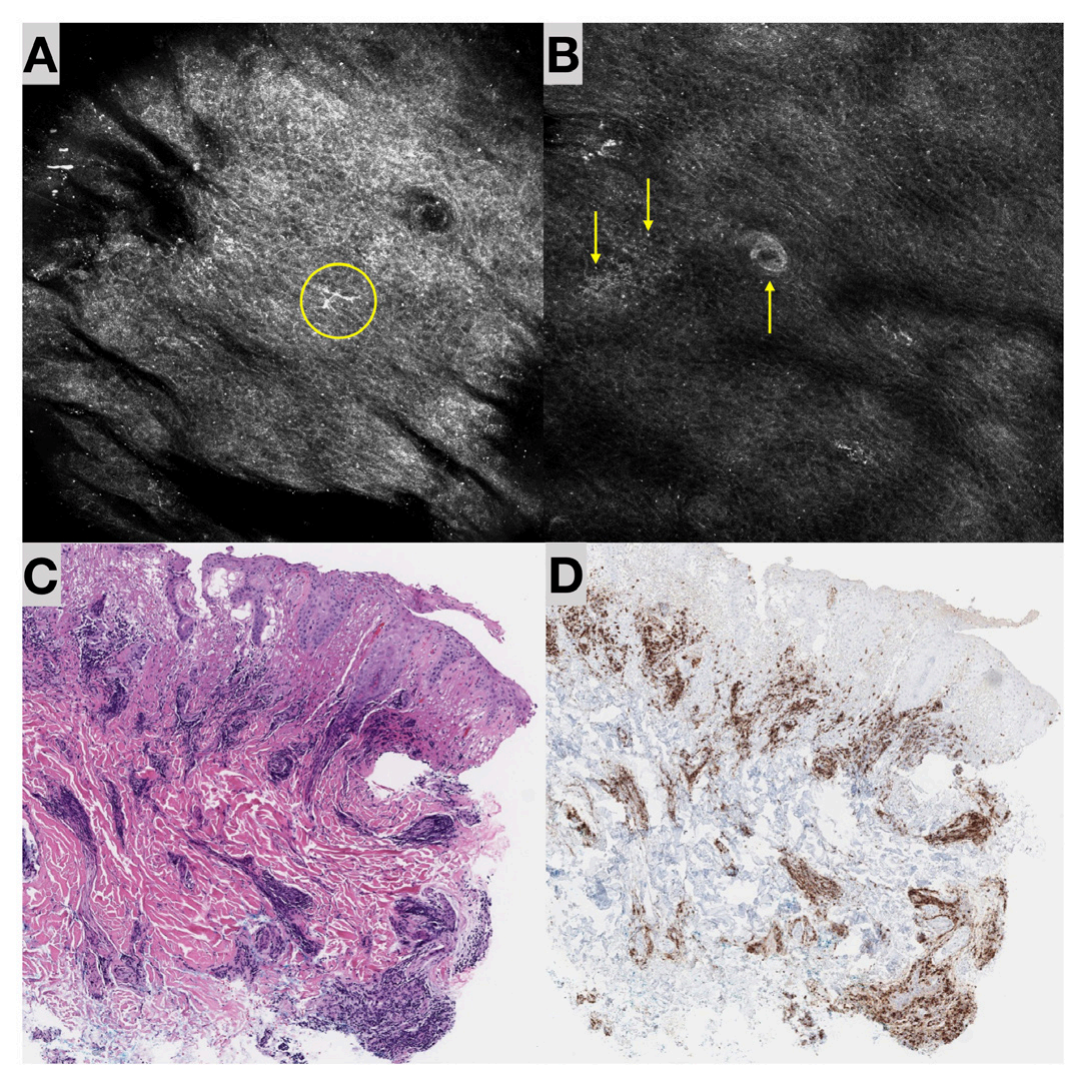
Biopsy imaging. ( **A**) Reflectance confocal microscopy (RCM) image (1mm x 1mm) of the biopsied toe showing an enlarged keratinocytes and presence of dendritic cells (activated Langerhans cells in the circle). ( **B**) RCM image (1mm x 1mm) of the epidermis and dermal epidermal junction showing bright white dots representing inflammatory cells (↑) and an enlarged, bright and reflective swollen dermal papillae (↓). ( **C**) Histopathology H&E image at 10x magnification. ( **D**) Histopathology H&E image at 10x magnification stained for CD8, which shows the vast majority of lymphocytes to be T cells with a relatively unremarkable CD4:CD8 ratio, present mostly in a perivascular distribution and in the superficial dermis.

A 3mm punch biopsy was taken on the patient’s third left lateral toe in the most prominent area of chilblains-like lesions findings approximately 2 weeks after the onset of lesions and during early stages of lesion resolution. Pathologic findings included parakeratosis, overlying vacuolar alteration of the basal layer with dyskeratosis, fibrinoid degeneration and edema of the papillary dermis ( Figure 2). No thrombi were seen. Superficial and deep perivascular lymphocytic infiltrate with extravasated red cells were also present with a final pathologic diagnosis of chilblains/perniosis, consistent with those seen in COVID-associated chilblains-like lesions (“COVID-toes”). Immunohistochemical analysis demonstrated the perivascular lymphocytic infiltrate to be composed predominantly of CD3+ T cells, with very rare CD20+ B cells. The T cells demonstrated an unremarkable CD4:CD8 ratio. Rare CD163+ histiocytes were scattered throughout.

An *in vivo* virtual biopsy with reflectance confocal microscopy (RCM; Vivascope 3000, Caliber I.D., Rochester, NY, USA) was performed on the same toe that was examined histologically. Findings included an irregular epidermis with broadened honeycombed pattern and absence of pagetoid cells. In the dermal-epidermal junction, enlarged and swollen highly reflective papillae as well as dendritic cells (activated Langerhans cells) and bright dots representing inflammatory infiltrate were present. Within two days of the biopsy and virtual biopsy, the patient was tested for SARS-COV-2 (PCR) and antibodies, neither of which yielded a positive result. The timing of the tests or failure of many to mount an antibody-mediated response make it difficult to determine whether this is a COVID-related case or not, since the specificity of available testing is low. No therapeutic intervention was made and upon follow-up the patients symptoms resolved.

Of interesting note, the patient reported that at the same time she and her husband developed the gastrointestinal symptoms, her elderly pet cat also developed an upper respiratory infection with a three-day history of lethargy, loss of appetite and increased sneezing and coughing, which spontaneously resolved. Additionally, the interior distal aspect of the cat’s ears developed multiple red-purple, well-circumscribed, slightly raised spots bilaterally that resembled the patient’s cutaneous findings. These lesions resolved within 3 weeks of onset.

## Discussion

Cutaneous lesions associated with coronavirus-induced vasculitis has been previously reported in a cat with feline infectious peritonitis and concurrent feline immunodeficiency virus infection ^4^. In this case, the animal presented with a similar two-week history of pyrexia, loss of appetite and weight loss, sneezing, bilateral nasal and ocular discharge to our patient’s cat. The cat described in this previous case report presented with multiple well-circumscribed slightly raised, red nodules and positive FCoV ^4^. Recently at the Bronx Zoo in New York, eight big cats (5 tigers and 3 lions) have tested positive for the COVID-19 virus and are believed to have been infected by an asymptomatic zookeeper. The cats started showing symptoms including a dry cough, with one tiger who did not show any symptoms for coronavirus ^5^. A recent publication also suggests that cats may be intermediate hosts of the SARS-Cov-2 ^6, 7^.

This is the first report of reflectance confocal microscopy findings in chilblains-like lesions and the large number of dendritic antigen-presenting cells may be a distinguishing feature in its presentation. Further studies are needed to determine if these RCM findings are exclusive to chilblains-like lesions, due to the negative COVID-19 result, or also found in traditional chilblains. It is not possible to conclude that our patient had COVID-19-associated chilblains-like lesions because she did not have a positive viral or antibody test and may be a limitation to our findings. However, she did have a pattern of development that is highly suspicious for infection-induced chilblains-like lesions, including a history of respiratory and gastrointestinal symptoms, aged below 50 years, no comorbidities, a previous state of good health, lack of prior history of skin findings consistent with chilblains, lack of cold temperatures in the region, latency between mild systemic symptoms and the morphology of chilblains-like lesions and the development of similar finding in her pet cat.

In a recent article the Hubiche T *et al.* reported positive COVID-19 serology in only 30% of patients who presented with chilblains-like lesions suggesting that chilblains-like lesions are associated with mild or asymptomatic SARS-CoV-2 infection ^8^. While our findings in combination may be coincidental, we hypothesize that there may be a delayed immune-mediated reaction to SARS-COV-2 in genetically predisposed patients who may test negative during a certain diagnostic window. Further studies are needed to determine the accurate diagnostic window for COVID-19 diagnosis and possible cutaneous manifestations.

## Consent

Written informed consent was obtained from the patient for the publication of this case report and any associated images.

## Data availability

All data underlying the results are available as part of the article and no additional source data are required.
