# Supplementary material for: Case Report: Chilblains-like lesions (COVID-19 toes) during the pandemic - is there a diagnostic window?
Source: F1000Res. 2020 Aug 26;9:668. Originally published 2020 Jul 2. [Version 2] doi: 10.12688/f1000research.24766.2 (PMC7459833; doi:10.12688/f1000research.24766.2)
Supplement: Peer review history

# Peer review: Reviewer response for version 2

**DOI:** 10.5256/f1000research.28794.r70283

**Published:** 2020-10-15

**Authors:** Marco Ardigo

**Affiliations:** 1 Clinical Dermatology, San Gallicano Dermatological Institute IRCCS, Rome, Italy

My comments on RCm evaluation are still not answered by the authors. I was waiting for clarification of the following points:

  

- Dermal features (important for diagnosis) cannot be seen on confocal microscopy. Please, clarify this point and indicate the effective role of RCM in the evaluation of the case.
- Why the authors underline the absence of pagetoid cells in a non suspected melanocytic lesion? I personally do not think that this is the correct confocal description as this is not a melanocytic lesion... the report of absence of pagetoid cells is not necessary.
- How the authors define activated Langerhans cells based on confocal microscopy observation alone?

 

 I'd love to receive comments on the following queries I already indicated in my first review.”

Are enough details provided of any physical examination and diagnostic tests, treatment given and outcomes?

Yes

Is the case presented with sufficient detail to be useful for other practitioners?

No

Is sufficient discussion included of the importance of the findings and their relevance to future understanding of disease processes, diagnosis or treatment?

No

Is the background of the case’s history and progression described in sufficient detail?

Yes

Reviewer Expertise:

NA

I confirm that I have read this submission and believe that I have an appropriate level of expertise to state that I do not consider it to be of an acceptable scientific standard, for reasons outlined above.

# Peer review: Reviewer response for version 2

**DOI:** 10.5256/f1000research.28794.r70284

**Published:** 2020-08-27

**Authors:** Adam Reich

**Affiliations:** 1 Department of Dermatology, University of Rzeszów, Rzeszów, Poland

None

Are enough details provided of any physical examination and diagnostic tests, treatment given and outcomes?

Yes

Is the case presented with sufficient detail to be useful for other practitioners?

Yes

Is sufficient discussion included of the importance of the findings and their relevance to future understanding of disease processes, diagnosis or treatment?

Yes

Is the background of the case’s history and progression described in sufficient detail?

Yes

Reviewer Expertise:

general dermatology

I confirm that I have read this submission and believe that I have an appropriate level of expertise to confirm that it is of an acceptable scientific standard.

# Peer review: Reviewer response for version 2

**DOI:** 10.5256/f1000research.28794.r70285

**Published:** 2020-08-27

**Authors:** Magdalena Żychowska

**Affiliations:** 1 Department of Dermatology, Institute of Medical Sciences, Medical College of Rzeszow University, Rzeszow, Poland

The revised version is improved and well-written.

Are enough details provided of any physical examination and diagnostic tests, treatment given and outcomes?

Yes

Is the case presented with sufficient detail to be useful for other practitioners?

Yes

Is sufficient discussion included of the importance of the findings and their relevance to future understanding of disease processes, diagnosis or treatment?

Yes

Is the background of the case’s history and progression described in sufficient detail?

Yes

Reviewer Expertise:

Dermoscopy, non-invasive skin imaging techniques

I confirm that I have read this submission and believe that I have an appropriate level of expertise to confirm that it is of an acceptable scientific standard.

# Peer review: Reviewer response for version 1

**DOI:** 10.5256/f1000research.27321.r66288

**Published:** 2020-07-30

**Authors:** Magdalena Żychowska

**Affiliations:** 1 Department of Dermatology, Institute of Medical Sciences, Medical College of Rzeszow University, Rzeszow, Poland

The authors presented an interesting case of chilblains-like lesions in a 48-year-old woman. The case report is well-organized and written in a clear fashion. Furthermore, it includes dermoscopic and confocal findings of chilblains-like lesions.

 However, I have several comments and suggestions:

- the abstract could be longer and should include more details regarding the presented case and findings
- if available, please include lab findings (antinuclear antibodies? serology for Mycoplasma/Chlamydia infection? C-reactive protein?)
- I'm not a big supporter of the acronym introduced by the authors just to separate the name of the dermatological lesions from chronic lymphocytic leukemia (CLL).

The dermoscopic and confocal findings presented by the authors may be of some value for future diagnostic process of COVID-19-related lesions, but it should be taken into consideration that the infection was not confirmed in the presented patient and the authors only hypothesize that there may be a delayed immune-mediated reaction. Undoubtedly, further studies, comparing the dermoscopic and confocal features of COVID-19 toes and chilblains-like lesions resulting from other conditions are needed.

Are enough details provided of any physical examination and diagnostic tests, treatment given and outcomes?

Yes

Is the case presented with sufficient detail to be useful for other practitioners?

Yes

Is sufficient discussion included of the importance of the findings and their relevance to future understanding of disease processes, diagnosis or treatment?

Yes

Is the background of the case’s history and progression described in sufficient detail?

Yes

Reviewer Expertise:

Dermoscopy, non-invasive skin imaging techniques

I confirm that I have read this submission and believe that I have an appropriate level of expertise to confirm that it is of an acceptable scientific standard, however I have significant reservations, as outlined above.

# Response

**Published:** 2020-08-12

**Authors:** Alexander Witkowski

**Affiliations:** Oregon Health & Science University, USA

To Reviewer Dr. Magdalena Żychowska: All comments addressed, the abstract has been made longer including details regarding the presented case and findings; lab findings unavailable ; CCLL has been removed and it remains Chilblains-like lesions ; new citation added (number 8) and added in the discussion.  Thank you for your comments and suggestions

# Peer review: Reviewer response for version 1

**DOI:** 10.5256/f1000research.27321.r66287

**Published:** 2020-07-27

**Authors:** Adam Reich

**Affiliations:** 1 Department of Dermatology, University of Rzeszów, Rzeszów, Poland

The authors demonstrated a case of chilblain-like lesions in a 48-year old woman. This case report is interesting, however, I have several comments, which can possibly be of relevance. Recently, "A COVID toe" has been described as a manifestation of COVID-19 infection. However, most reports are focused on single patients or demonstrate only a case series. Today, it is a matter of debate, whether indeed this is a specific cutaneous manifestation of COVID-19 infection or whether it may also result from other viral infections. In a recent study by Hubiche et al. (Hubiche T et al. Negative SARS-CoV-2 PCR in patients with chilblain-like lesions [published online ahead of print, 2020 Jun 18]. *Lancet Infect Dis*. 2020;S1473-3099(20)30518-1 ^1^) in only 30% of patients, COVID-19 infection (current or past) could be proofed. It is also important to exclude other causes of chilblains, mainly lupus erythematosus, which is not documented in current report. 

 The authors also propose a new acronym for chilblains-like lesions with an additional word cutaneous (CCLL) to distinguish it from CLL - chronic lymphocytic leukemia. However, I disagree with such a proposal as adding a word "cutaneous" implicates that there are also extracutaneous manifestations of chilblains-like lesions, which is not the case in the current situation.   

 Some minor comments:

 The authors should avoid to use the term "unique" in the abstract

 It is unclear, what the authors meant under the term "blister-like lesions"

Are enough details provided of any physical examination and diagnostic tests, treatment given and outcomes?

Yes

Is the case presented with sufficient detail to be useful for other practitioners?

Yes

Is sufficient discussion included of the importance of the findings and their relevance to future understanding of disease processes, diagnosis or treatment?

Yes

Is the background of the case’s history and progression described in sufficient detail?

Yes

Reviewer Expertise:

general dermatology

I confirm that I have read this submission and believe that I have an appropriate level of expertise to confirm that it is of an acceptable scientific standard, however I have significant reservations, as outlined above.

## References

[1] : Negative SARS-CoV-2 PCR in patients with chilblain-like lesions. *The Lancet Infectious Diseases*.2020; 10.1016/S1473-3099(20)30518-1 10.1016/S1473-3099(20)30518-1 32563281PMC7302762

# Response

**Published:** 2020-08-12

**Authors:** Alexander Witkowski

**Affiliations:** Oregon Health & Science University, USA

To Reviewer Prof. Adam Reich: All comments addressed, the term "unique" in the abstract has been removed ; "blister-like lesions" have been changed to blisters as this was the actual presentation ; CCLL has been removed and it remains Chilblains-like lesions ; new citation added (number 8) and added in the discussion.  Thank you for your comments and suggestions

# Peer review: Reviewer response for version 1

**DOI:** 10.5256/f1000research.27321.r66284

**Published:** 2020-07-17

**Authors:** Marco Ardigo

**Affiliations:** 1 Clinical Dermatology, San Gallicano Dermatological Institute IRCCS, Rome, Italy

This is a report of a case of chilblains-like lesions on a COVID-19+ patient.

 Even if the case could be potentially interesting (several cases have been already reported in the literature) and it has been well presented, the use of confocal microscopy in this specific case does not add any new information with no distinctive features.

 Few points:

- Dermal features (important for diagnosis) cannot be seen on confocal microscopy.
- Why the authors underline the absence of pagetoid cells in a non suspected melanocytic lesion?
- How the authors define activated Langerhans cells based on confocal microscopy observation alone?

In my view, this case report does not merit to be considered for indexing as it is presented.

Are enough details provided of any physical examination and diagnostic tests, treatment given and outcomes?

Yes

Is the case presented with sufficient detail to be useful for other practitioners?

No

Is sufficient discussion included of the importance of the findings and their relevance to future understanding of disease processes, diagnosis or treatment?

No

Is the background of the case’s history and progression described in sufficient detail?

Yes

Reviewer Expertise:

Rare skin diseases and non invasive techniques for skin analysis

I confirm that I have read this submission and believe that I have an appropriate level of expertise to state that I do not consider it to be of an acceptable scientific standard, for reasons outlined above.
